# Ethnography, ethnobiology and natural history: narratives on hunting and ecology of mammals among *quilombolas* from Southeast Brazil

**DOI:** 10.1186/s13002-020-0359-3

**Published:** 2020-02-21

**Authors:** Helbert Medeiros Prado, Raquel Costa da Silva, Marcelo Nivert Schlindwein, Rui Sérgio Sereni Murrieta

**Affiliations:** 1 Centro de Ciências e Tecnologias para Sustentabilidade , Universidade Federal de São Carlos, João Leme dos Santos Highway 110 km, Sorocaba, SP, 18052-780 Brazil, Universidade Federal de São Carlos, João Leme dos Santos Highway 110 km, Sorocaba, SP 18052-780 Brazil; 2 Centro Nacional de Pesquisa e Conservação de Mamíferos Carnívoros, Instituto Chico Mendes de Conservação da Biodiversidade, 8600 Hisaichi Takebayashi Highway, Atibaia, SP 12.952-011 Brazil; 3 Instituto de Biociências, Universidade de São Paulo, 277 Matão Str, São Paulo, SP 05508-090 Brazil

**Keywords:** Ethnosciences, Environmental Anthropology, Hunting, Ethnography, Theory of Practice; *Quilombolas*, Atlantic Forest

## Abstract

**Background:**

As a leading practice of *Homo sapiens*’ environmental experience for hundreds of millennia, hunting continues to evoke key research inquiries in the fields of archaeology, human ecology, and conservation biology. Broadly speaking, hunting has been mainly a subject of qualitative-symbolic and quantitative-materialistic schemata of analyze, among anthropologists and biologists, respectively. However, the phenomenological dimension of the hunting experience, in the course of individuals` everyday life, received little academic attention until this century. This study analyzes the daily praxis of hunting among *quilombolas* (descendants from runaway African slaves) in Southeast Brazil, making use of an ethnographic approach of phenomenological orientation, which dialogue with central ethnobiological issues. The authors also report the local ecological knowledge about mammals hunted in the area, and its relationship to the scientific literature on this subject.

**Methods:**

Between 2016 and 2019, the authors made use of participant observation and informal interviews among eight key local participants, in three *quilombola* communities in the Ribeira Valley (São Paulo, Brazil). Fragments of authors’ field notes and parts of interviewers’ speeches make up the core results obtained.

**Results:**

Articulating local knowledge to scientific literature, this study yielded a hybrid and comprehensive narrative about natural history of the mammals in the area. The authors also accessed elementary aspects of research participants’ experience in hunting, such as strategies, tactics, motivations, and feelings. They reveal a set of human behavior dispositions that seems to emerge only in the context of the action, modulating the praxis of hunting on the course of individuals’ everyday life.

**Conclusion:**

Ethnography, ethnobiology, and natural sciences backgrounds were systematically articulated in this research. This made possible to get a contextualized and multifaceted understanding of hunting praxis in the Ribeira Valley, an important socioenvironmental context of Atlantic Forest in Brazil. The role of an ethnographic approach applied to ethnoecological and biological conservation issues is especially considered here.

## Background

In the field of natural sciences, especially applied ecology, the practice of hunting is generally addressed through quantitative analysis, comparing the numbers of a species slaughtered with their reproductive potential and resilience [1–4]. These studies produce future prognoses that help develop strategies for the long-term maintenance of species being exploited [5, 6], as well as the biota and environmental services indirectly impacted by defaunation [7].

In social sciences, hunting is an important topic in anthropological studies. In the context of lowland South America, especially the Amazon, ethnographies of hunting among native peoples have made significant contributions to recent anthropological debates, especially with regard to ontological issues [8]. Examples of these contributions include the systematization of animism [9, 10] and the formulation of an Amerindian perspectivism [11, 12], among others [13, 14].

As a complement to the two approaches mentioned above, studies on hunting can also focus on the local body of knowledge about the natural history of the species being examined, taking an ethnobiological approach informed by an ethnographic orientation [15, 16]. By gaining access to the wealth of practical knowledge about local fauna, ethnobiological studies focused on hunting can also make it possible to record local repertoires that are of interest to zoology, ecology and conservation. Ethnoecology—considered here as part of the broader field of ethnobiology—has shown great potential for revealing landscape use patterns [17, 18], historical fluctuations in abundance [19, 20], and cryptic behaviors among vertebrates not yet well known in the scientific literature [21, 22].

This article presents a set of local narratives about the hunting and natural history of medium- and large-sized mammals. These narratives will be considered in relation to both the zoological and anthropological literature on the topics presented. Activity pattern and spatial-temporal dynamic of mammals in the area, use of dogs in hunting, and aspects of behavior of ungulates and primates during hunting activities are some ecological topics addressed by this work.

This paper also uses a phenomenological orientation [23, 24] to present and discuss microaspects [25] related to the daily hunting routine, individual motivations, and the affective and mythic dimensions of this activity. Recognition of the many layers of meaning in the praxis of hunting can have important implications for the development and effectiveness of management and conservation plans for the species concerned.

## Methods

### Ribeira Valley and its quilombola communities

Ribeira Valley is located along southeastern São Paulo state and northeastern Paraná state, occupying an area of 2,830,666 ha [26] and forming part of the largest continuous area of Atlantic Forest in Brazil, which has been designated by UNESCO as a world heritage site. The region’s climate is hot and humid [27], and its predominant vegetation is classified as Montane/Submontane Dense Ombrophilous Forest [28].

The remaining *quilombola* communities of Ribeira Valley trace their origin to slaves who escaped or were freed or abandoned during the Brazilian slavery colonial regime in the mid-18th century [29]. Since their establishment, *quilombolas* have relied on slash-and-burn (or coivara) agriculture for subsistence, together with hunting and raising pigs and chickens [30].

The formation of these rural communities in the first half of the 19th century coincided with the accelerated production and trade of rice throughout Ribeira Valley [31]. This period appears to have had a decisive influence on the agricultural practices and local and regional trade relations experienced by these populations during the early decades of the 20th century [30].

The regional production cycles with the greatest impact on the *quilombolas* of mid-Ribeira Valley have been the extraction and trade of heart of palm (*Euterpe edulis*), which gained strength starting in the 1960s [32]; the intensification of banana production, especially starting in the 1970s [33]; and the cultivation of peach palm (*Bactris gasipaes*), which has gained regional prominence over the past 15 years [34].

**Fig. 1:**
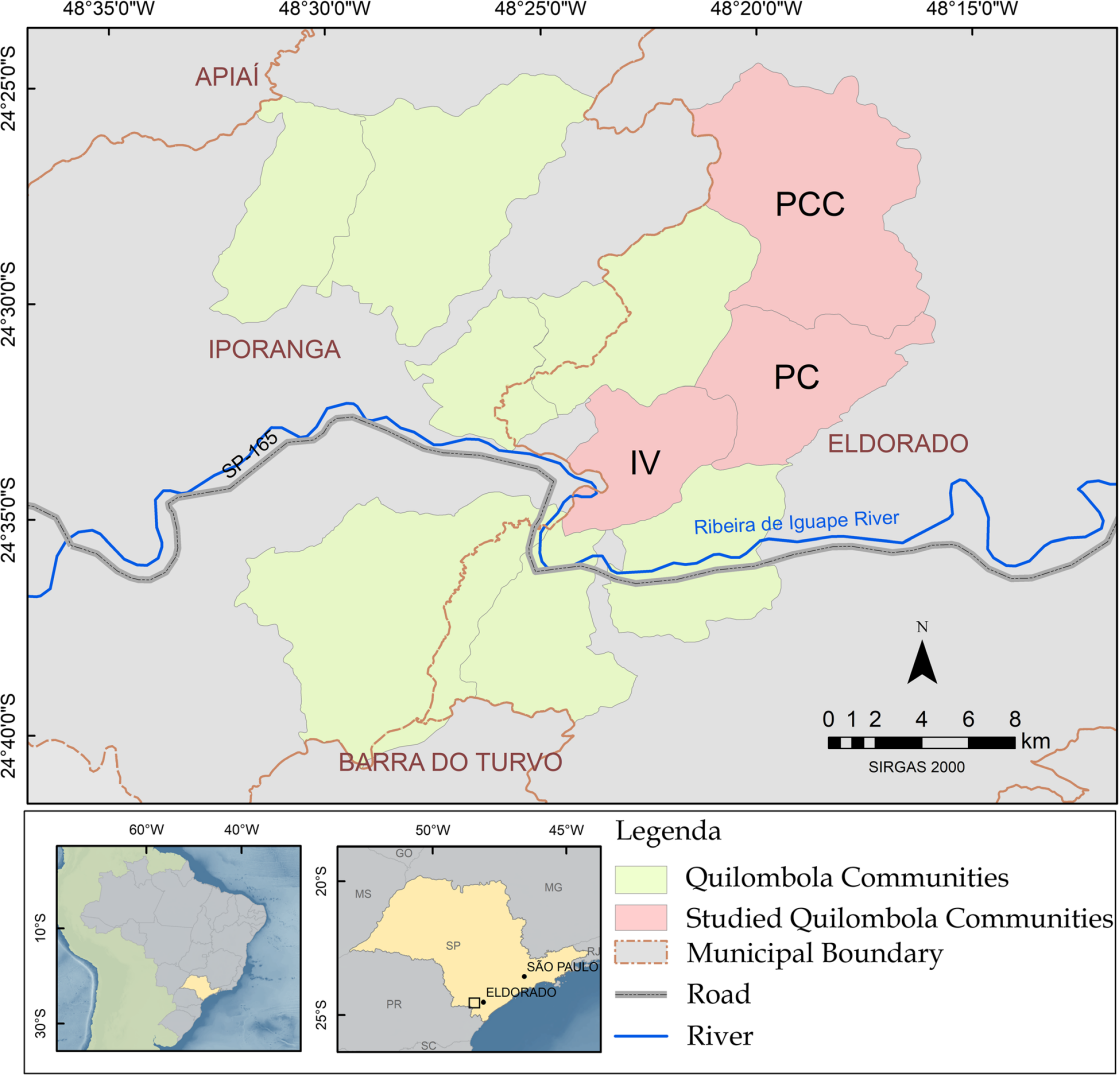
Studied *quilombolas* communities and surrounding area in the Ribeira Valley (São Paulo, Brazil). IV, Ivaporunduva; PC, Pedro Cubas; PCC, Pedro Cubas de Cima (Elaborated by Camila Barbosa)

The three communities that participated in this study were Ivaporunduva, Pedro Cubas, and Pedro Cubas de Cima (Fig. 1). Pedro Cubas covers 3806 ha, with a population of approximately 150 people and 40 households [35; 2005 data]. Pedro Cubas de Cima has a recognized area of 6875 ha. Its population of approximately 120 is distributed among some 30 households [35; 2005 data]. With a recognized area of 2,754 ha, Ivaporunduva has approximately 320 inhabitants and 80 households [35,36; 2005 data).

The territory of the three communities studied is composed primarily of native mature and secondary forests in various stages of regeneration (90–95% of the area), with 5–10% made up of pastures and other landscape features such as waterways and roads [35, 2007 data]. Most of the population is made up of family farmers. Other strategies of production include forest extraction and subsistence hunting. In addition to trade in agricultural products, other sources of income include government income-transfer programs, day labor, and retirement pensions [35, 36].

### The ethnographic approach

The content of this article is the outcome of the authors’ work in two independent projects. One project focused on a survey of medium- and large-sized mammals in the Ivaporunduva area, combining ecological and ethnoecological approaches [37]. The second project, which is still ongoing, is concerned with the various practices by which the *quilombola* families of the region use landscape resources [38]. Both authors used the participant observation technique combined with informal interviews in their research [39]. The authors made audio recordings of some of their interviews and kept systematic field journals. Through this ethnographic approach, local narratives of hunting, natural history, and mammal ecology were gathered and will be analyzed here.

Some of this article's results are the product of HMP’s participation in the daily activities of Pedro Cubas and Pedro Cubas de Cima, especially those involving hunting and the preparation and consumption of the slaughtered animals. Other results were generated by RCS’s participation in expeditions into the forest in Ivaporunduva, accessing the *quilombolas*’ knowledge about potential areas used by different species. In both studies, the informal interviews were conducted in conjunction with the practice of participant observation [39].

The use of audio recorders was not prioritized while informal interviews were being conducted due to the spontaneous nature of this format; recorders might interrupt the narratives’ flow and cause some degree of discomfort to those being interviewed. In some situations, however, when a sufficient degree of trust had been established between the parties and the narrative was long, audio recording was used, always with the interviewee’s permission.

In general, however, the primary method of recording local experiences and accounts was the authors’ systematic use of field journals. The authors also wrote down notes whenever possible or considered relevant: during short pauses between different activities or even during the course of an activity or conversation (and not only at the end of the day). When the author was better acquainted with the interviewees, it was even possible to reproduce short passages of the account while the narratives were being told. Some of these passages are included in the results presented here.

The set of techniques and procedures mentioned above were used by the authors in light of their interest in capturing the processual quality of the daily practices and local knowledge involved. The authors therefore adopted an ethnographic approach with a phenomenological orientation [24, 40]. This allowed accessing the continuous flow of the individuals’ experiences in his or her environment (such as the praxis of hunting) as well as the flow of individuals’ narratives that emerged from the ethnographic experience [23].

The data are presented in two ways in this article: (1) reproduction of the participants’ narratives, right indented and with quotation marks, and (2) passages from the authors’ field journal, right indented and without quotation marks. These passages from the field journals also contain short quotations of local residents’ speech, which also appear in quotation marks. Translations of local terms appear right after the term in brackets. When local concepts require explanation, these appear in footnotes.

This article contains narratives of two residents of Pedro Cubas: Mota (age 65) and Edivan (age 30); three residents of Pedro Cubas de Cima: Mauro (age 62), Zeca (age 58) and Duda (age 50); and three residents of Ivaporunduva: Renato (age 72), Danilo (age 65) and João (age 42). The study’s participants were selected based on the authors’ prior experience in the communities and their knowledge of the various individual’s profiles [41]. Priority was given to residents with extensive experience in traditional practices of landscape resource use who were willing to participate in the study. Participants were also selected through an adaptation of the snowball method [42], in which experienced residents who understand the study’s purpose indicate other residents whose profiles suit the study. The gender bias is the result of men’s greater involvement in hunting activities in this ethnographic context than women [22].

The two research projects on which this article is based were presented to the participating communities prior to their commencement. The study underway in Pedro Cubas and Pedro Cubas de Cima was authorized by the Research Ethics Committee of Federal University of São Carlos [*Universidade Federal de São Carlos*, *UFSCar*]. The study in Ivaporunduva followed the norms of the American Anthropological Association’s code of ethics [43]. The three communities also authorized the research project through the Free and Informed Consent forms signed by their legal representatives. Fictitious names were used to protect the identities of study participants. It is worth mentioning that the subsistence hunting of wild species is legal in Brazil when it is done by traditional populations, which is the case for the communities studied [44].

### Faunistic group mentioned in the local narratives

The locally collected narratives refer to hunting events and ethnozoological repertoires involving medium- and large-sized mammals. This faunistic group carries out important ecological functions, such as controlling prey populations and shaping vegetation dynamics via seed dispersal and predation, seedling predation, and among other interactions [7, 45–47]. This fauna also represents a significant source of calories and protein for neotropical rural populations in general [3, 48, 49], including inhabitants of the Brazilian Atlantic Forest [50].

## Results and discussion

A variety of hunting methods is found in the Ribeira Valley. Hunting in the studied communities is mainly nocturnal, when individuals wait for their prey in a “trepeiro.” “Trepeiro” consists in an artisanal structure made of wood set high in the trees to accommodate the hunter at a strategic location for hunting. Commonly, the local is prepared previously with “ceva,” a bait used by *quilombolas* to attract game animals. Banana bunch and rock salt are the most commonly used bait for animals in the area. This type of hunting is performed individually, with the use of flashlight and shotgun, and the animals slain are mostly paca (*Cuniculus paca*), deer (*Mazama sp*.), and occasionally lowland tapirs (*Tapirus terrestris*).

Another method of hunting in the area is a diurnal walking tour in the forest, with the use of hunting dogs. This practice can be done individually or by groups of two or more people armed with shotgun. Deer, peccary (*Pecari tajacu*), coati (*Nasua nasua*), and monkeys (i.e., *Alouatta guariba*) are commonly slaughtered with this method. Artisanal traps made of woods are also used by local residents. They are set up preferably in fallows (secondary forests) near to the village. Animals captured with this method are small preys such as armadillos (*Dasypus sp*.) and big-eared opossum (*Didelphis aurita*).

The 13 mammals mentioned in the local narratives were deer (a local term that includes the two most common cervids in the area, *Mazama americana* and *Mazama gouazoubira*, as well as *Mazama nana* and *Mazama bororo*, which may also be found in the region), peccary (*Pecari tacaju*), paca (*Cuniculus paca*), coati (*Nasua nasua*), fox (*Cerdocyon thous*), raccoon (*Procyon cancrivorus*), puma (*Puma concolor*), jaguar (*Panthera onca*), howler monkey (*Alouatta guariba*), and southern muriqui (*Brachyteles arachnoides*). For the remainder of the article, the species will be referred to only by their common names.

### Animal activity pattern, lunar cycle, and hunting strategies

"Pacas come out at night, and you're more likely to see them during the waning moon, three days after the waxing moon" (João, age 42, RCS's field journal, 05/25/2017).

We start the discussion of local narratives with this account that summarizes two aspects of the ecology of a particular species, the paca. Here, we can access a local resident’s perception of the species’ activity period (nocturnal), and more particularly, an indication that the animal is more active (“you’re more likely to see them”) in a certain lunar phase (waning) associated with less light.

This information is associated with the concepts of photophobia in general and of lunar phobia in particular, a behavior attributed to species as an antipredator strategy. It has also been suggested that pacas exhibit photophobia in settings such as open fields [51] and secondary forests [52] in the Amazon, and it has been shown in an Atlantic Forest fragment in southern Brazil [53]. In primary forests in the Amazon, however, Michalski and Norris [54] did not observe a clear correlation between lunar phases and the occurrence of pacas in samplings using camera traps.

There appears to be an interaction between luminosity and habitat types. Because their canopies are less dense, secondary forests allow greater light penetration, while primary forests permit less light to penetrate. It is thus to be expected that photophobia would have a greater impact in secondary forests and, therefore, that it would be more likely to be recorded. We know that the areas most used by the *quilombolas* for hunting and other activities are secondary forests [41], and this is in keeping with the local perception that pacas avoid the nights with the brightest moonlight in this setting.

**Fig. 2:**
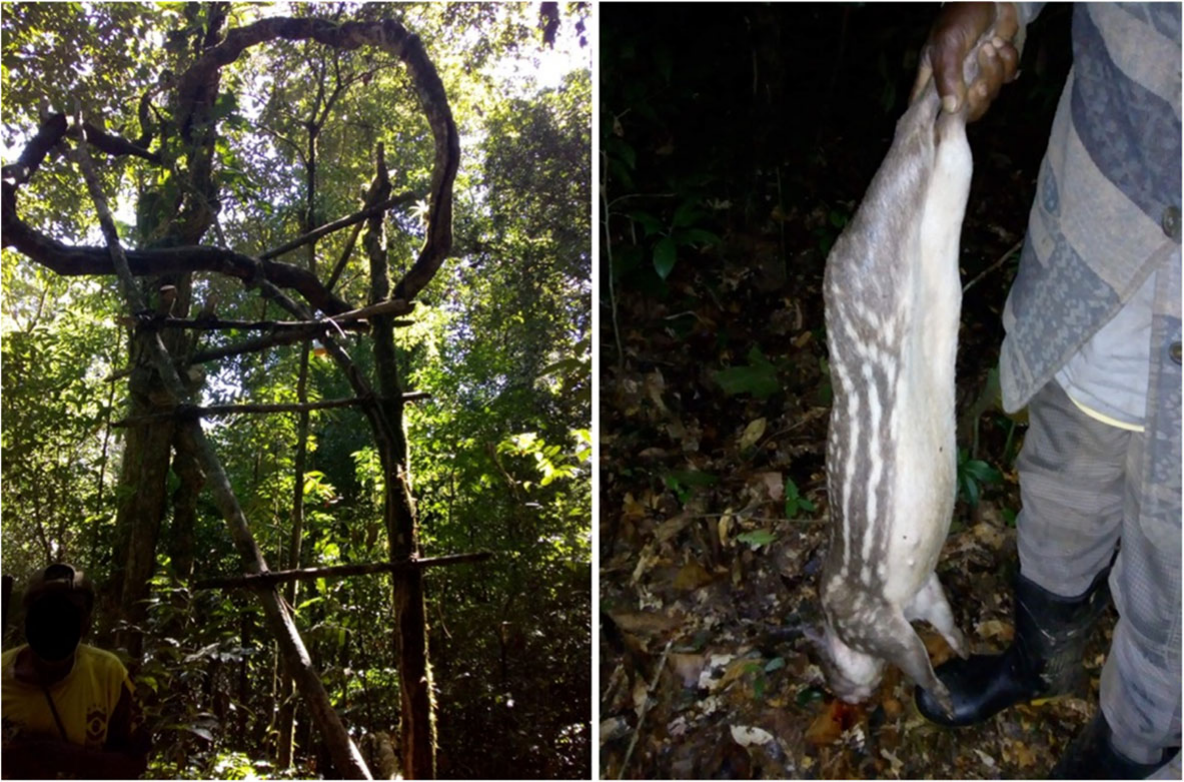
Left: “Trepeiro” (treestand) used in the two hunting events reported by HMP in the text (Photograph: 08/12/2019). Right: Paca (*Cuniculus paca*) slaughtered during the hunting event of 07/18/2019, reported by HMP in the text (Photograph registered by HMP)

The experience of one of the authors (HMP) with local hunters (Fig. 2), including waiting for long periods in a “trepeiro” provided more details about the activity patterns of nocturnal species in the region with regard to lunar phases.

Today, I arrived at Mauro’s (age 62) house in the early afternoon. During our conversation, he said that he was going to visit a "ceva" in a nearby “capoeira”^1^ between late afternoon and early evening. I asked if I could go along with him. He agreed, but warned me that we would have to go early. "It's a full moon tonight, and the deer is very agitated at the beginning of the night." Mauro explained that after the moon "comes out," the deer stop moving and do not come to the "ceva" anymore. Mauro said the moon would "come out" around 8 pm this evening. He also reported that the deer had been visiting the location and licking the salt for the past two days. We arrived at the location shortly before 6 pm. By around 6:30 pm, it had grown dark. We climbed into the “trepeiro” and remained there in complete silence. Twice we heard what sounded like a large animal coming down the path near the "ceva." But the animal didn't stop to lick the salt or touch the banana. I wondered what it could be. Mauro thought it was the same deer who had been visiting the "ceva" the previous days. Somehow, the deer "suspected" our presence, he said. Between 7:30 and 8:00 pm the forest slowly grew lighter, until it was brightly lit by the full moon, which was already visible in the sky. It was time to go home (HMP’s field note entry, 07/16/2019).

Late in the afternoon today (07/18/2019), after a brief drizzle, we were able to go up the path again toward the same location. Mauro said we wouldn't have to arrive at the “trepeiro” as early as we had the first night. Because we were already in the waning moon, the moon would "come out" around 9 pm, an hour later than it had two nights ago. In Mauro's judgment, animals like the deer and paca become active a little later, as they have more time to move about at night. Anyway, we arrived at the “trepeiro” around 6 pm. Between 6:30 and 7 pm, the forest was completely dark. I couldn't even make out the shape of the nearest trees. We could hear the sounds of fruit dropping onto the forest floor, the flutter of a bat's wings above us, and the rustling of a small animal circling the "ceva." At one point, Mauro pointed his flashlight toward the bananas. It was only a small marsupial. But it wasn't long before we heard the noise of a larger animal approaching. After a few minutes, the animal was already in the "ceva." Holding the flashlight with his left hand, Mauro shined the beam of light directly at the animal - it was a large paca eating the banana. Shotgun in his right hand, the aim, and the accurate shot. It was a male. Ecstatic, Mauro describes the animal's behavior before it arrived at the "ceva"; he talks about the animal's "cunning" and "suspicion" as it approached and drew away from the bait before finally stopping at it (which I would not have noticed). The kill happened around 7:30 pm, and we stayed there until around 9 pm. But no other animals came to the "ceva," and the moon "came out." Although there was not much light, at least the complete darkness was dispelled, and we could make out the shapes of some of the trees. Mauro said it was time to go home. "It's no use staying any longer, no more animals will come back to the ceva." (HMP's field journal, 07/18/2019).

The knowledge of hunters like Mauro suggests that the influence of the moon’s brightness on the activity pattern of nocturnal species may have a stronger influence on the time of activity rather than on whether the animal will be active or not on a particular night. This has received little attention in studies on the topic. The indication found in Mauro's narrative is in line with the study carried out by Michalski and Norris [54] in the Amazon, which did not find a significant relationship between the occurrence of pacas in their samplings and lunar phases (in contrast to the other studies mentioned above). The same study, however, found that in brighter nights, pacas tend to concentrate their activities just after sundown [54] or “before the moon comes out,” if we use Mauro's words to interpret this type of data.

In contrast to Mauro, who believes the moon’s light has a distinct influence on the times when animals are active, Michalski and Norris [54] found only a weak correlation between the moon’s luminosity and the times when pacas are active. This difference in magnitude may be due to the fact that those authors sampled a primary forest, while Mauro was hunting in a secondary forest approximately 30 years old with some openings in the canopy, and his “trepeiro” was located just a few meters from a one-meter-wide path. Under these circumstances, the brightness of the moon seems to have a greater impact on the nocturnal habits of the nocturnal prey that forage there. The *quilombolas* have also mentioned the impact of lunar phases on the olfactory signals left by peccaries in the forest, with implications for strategy of hunting with dogs. This topic will be introduced in the next section.

In addition to the interaction between local and scientific knowledge, the ethnographic experience summarized above may also reveal an affective dimension to Mauro’s involvement with hunting. Mauro lives alone, several kilometers outside the community, and the daily hunt is his main source of animal protein. He is now retired and owns a house in Vila do Batatal (near São Paulo State Highway 165, see Fig. 1), which could provide him a diet less reliant on hunting, if he preferred.

What seems to be at stake, however, is what we could call an ontological identification, a predisposition to behavior or an acquired taste associated with this practice, nourished, and fulfilled over the course of his daily life [23, 55, 56]. In fact, the many occasions on which HMP accompanied him on forest excursions were always filled with enthusiastic accounts of hunts in the distant or recent past; his memories were refreshed by passing specific places in the landscape associated with those episodes. This affective dimension of hunting is also associated with a form of social capital [57]. Mauro often shares meat he has hunted with some community members (especially family members) and is recognized by others as the most skilled hunter ever to live in the area.

### Olfactory cues in the forest and the use of dogs in hunting

In this section, we analyze the intricate interaction, and communication, between wild ungulates, hunting dogs, and hunters. To this end, we will articulate local narratives to zoological and anthropological literature on this topic. Firstly, we will retake the case of peccary’s olfactory signals and hunting with dogs, which we have briefly mentioned in the previous section. Below, we reproduce a section of one of the authors’ field journal. The account was recorded by HMP in the context of a conversation about hunting knowledge and techniques adopted in the region:

With regard to the peccary, Edivan (age 30) said that starting with the new moon, the peccary's "stink" [from the scent gland located on the animal's back] increases significantly, so that as the peccary brushes against the foliage while running from a dog, it leaves a strong scent, making it easier for the dog to track it (HMP’s field journal, 01/30/2019).

The passage cited above relates to another account recorded by HMP approximately 2 months earlier in another community in the same region. The conversation dealt with hunting activities in general:

Talking about the use of dogs in hunting, Zeca (age 58) stated that the dog "runs" [pursues] deer with its nose to the ground. "The deer gives off a secretion from between its toes." This speech suggests that a deer leaves an olfactory cue in its tracks. When the dog runs a "tateto" [peccary], it keeps its nose up because the "stink" is on the leaves (HMP’s field journal, 12/07/2018).

The two accounts cited above indicate differences in the modes by which peccaries and deer use scent in marking (as a form of social communication). These cues are used by hunting dogs. The peccary is known to have a dorsal scent gland located between 10 and 15 cm below the base of its tail [58, 59]. It is also known that deer and other cervids have tarsal and interdigital glands on their four legs that are associated with olfactory communication between individuals [60, 61].

Precisely because the peccary’s gland is located on its back, its odors are left on the leaves and branches the animal brushes against as it moves. In the case of *Mazama*, these olfactory cues remain in their tracks. In this sense, the account cited above also indicates that a dog pursues a deer with its snout to the ground but keeps his head up while chasing a peccary.

Thus, the position of the dog’s head during the chase can alert the hunter to the type of prey being pursued. This passage, in particular, indicates the great complexity involved in the interaction and communication between a hunter and his dog. This intimate and ancient bond between man and dog [62–64] is an element without which the local praxis of hunting cannot be well understood, nor its impact on the local fauna [65, 66]. The man-dog-deer relationship in the context of hunting will be explored in detail below.

In another conversation about hunting deer, Mauro (age 62) and Duda (age 50) described a technique used in the past, which was to release dogs to chase deer and then wait for the deer in a nearby stream or river:

“The deer comes down to the river, that's its only defense, just as a peccary burrows into a hole“ (Duda, HMP’s field journal, 12/29/2018).

According to these locals, when the hunter hears the deer approaching, he positions himself in the middle of the nearest stream and waits without moving, his machete at the ready. When the deer passes close to the hunter without noticing him, he strikes straight at the animal's neck.^2^ They also recounted that in one of these episodes, sports hunters from outside the community killed more than six deer in a single afternoon (HMP’s field journal, 12/29/2018).

The effectiveness of hunting deer with trained dogs [65, 66] is evident in the passage above. In this same interview, Mauro and Duda said that when hunters sight deer tracks and release their dogs to chase it, they already know which direction the deer will flee, based on the “spine” [hillslope] in that location. Still regarding deer hunting:

Mr. Mota (age 65) said that "in the river, the deer gets a bit disoriented," and that once he struck one with a machete [indicating the same tactic described above]. Edivan also confirmed that he waits for the deer in the river, but that he shoots it rather than using a machete. He also said that when it has run far, the deer arrives at the river very tired and also "a bit disoriented" (HMP’s field journal, 01/03/2019.

The study by Bateson [67] on behavioral and physiological aspects of *Cervus elaphus* killed with and without the aid of dogs in the UK is informative in this regard. Based on analysis of blood and muscle tissue samples collected at the moment of death, Bateson and Bradshaw [68] show that these cervids experience extreme physiological and psychological stress and extreme physical exhaustion during their flight. Significantly depleted blood sugar levels, muscle tears, damaged red blood cells, and extremely high cortisol levels were recorded in animals killed following short and long chases, which lasted, on average, for 3 h [68].

It is worth noting that the use of water courses by cervids is known in both the zoological and ethnographic literature [65, 66, 69, 70]. The search for rivers or streams during flight seems to be relatively effective at interrupting the chemical cue left by their tracks (given the presence of the interdigital glands mentioned above).

In a study of the Paranapiacaba mountain range, which includes Ribeira Valley, Vogliotti [[21], p., 20] also collected reports about this strategy in which deer flee from dogs in the direction of streams. Through sampling with camera traps, the same author showed that *Mazama bororo* was found with greater frequency in streams than in other landscape features such as forest paths, groves of fruit trees, or previously selected trails or latrines [[21], p., 38].

Following the passage cited above:

Edivan (age 30) reports that he typically releases the dog when he spots deer tracks. With each bark from the dog, he gauges his position with respect to the deer on a given forest "spine" [hillslope]. He also says the deer doesn't move until the dog "gets very close to him." He then bolts at high speed in the direction of the stream or river (HMP’s field journal, 01/03/2019).

Edivan continues saying that when the deer "is coming down the river sort of disoriented," he (Edivan) startles the animal as a tactic (causing it to freeze for a few seconds), then he shoots it (HMP’s field journal, 01/03/2019).

Edivan’s account is explicit and credible. His gestures seek to recreate his experiences during the hunt, and they are an important component of his narrative. The strength of his account suggests an intimacy with this activity and a special taste for storytelling. One would not expect to find this depth of involvement in (and knowledge about) a traditional practice in someone of Edivan’s generation—according to the general trend of intergenerational erosion of traditional knowledge in these communities [41], based on quantitative analytical studies. In this sense, Edivan’s accounts reinforce the special role of the ethnographic method with a phenomenological orientation [24, 40] and its ability to tease out personal singularities to complement the general view that an analytical method with a naturalist orientation would bring to this type of study.

It is important to remember that this account of the deer’s reaction when found by the dog and then by the hunter in the middle of a stream or river reflects the typical antipredator behavior of cervids, which is widely known in the zoological and ethological literature [71, 72]. When they sense a predator’s presence, these animals use the tactic of freezing to avoid being perceived and can remain frozen in place after they have been detected by the predator in order to assess the predator’s behavior. The prey then waits for the best moment to flee for safety, if necessary.

This behavior has been interpreted by biologists as advantageous to the prey, as it is a way to avoid wasting energy on premature and, perhaps, unnecessary flight [73]. In the specific case of the account cited above, this adaptive behavior in the deer proves effective with regard to its encounter with the dog, as the deer is able to reach the stream, even if exhausted.

However, upon the deer’s arrival at the stream, a second step of the hunt is initiated, as the deer encounters a second predator: the waiting hunter. When startled by the hunter in the stream (in the case of the account cited here), the deer reproduces its antipredator behavior of freezing. However, as precisely noted by Ingold [74] in a study of caribou (*Rangifer tarandus*) hunters in northern Finland, when the predator is a human able to kill at a distance, this behavior is no longer advantageous to the animal. This is because the hunter takes advantage of the moment the deer stands still to shoot it from a distance [23].

Last, the cervid behavior of freezing at the time of the hunt, with or without dogs, deserves a small ethnographic note. It is interesting to note, as Ingold [23] has also suggested, how this antipredator behavior among cervids seems to elicit, among hunting peoples, the conception that the animals are offering themselves to the hunter. Using ethnographies written about the Cree hunting people of northeastern Canada as an example and focusing on their relation with the caribou (*R*. *tarandus*), Ingold states:

“They (the Cree) say that the animal offers itself up, quite intentionally and in a spirit of good-will or even love toward the hunter. The bodily substance of the caribou is not taken, it is *received*. And it is at the moment of encounter, when the animal stands its ground and looks the hunter in the eye, that the offering is made.“ (Ingold [23], p.13).

It is worth mentioning that the model of an offering involving hunting has been widely discussed in anthropological literature, as have the logics of reciprocity and predation, to cite the Amazon case [75]. This ontological level of analysis, with regard to how local residents of Ribeira Valley (São Paulo) might explain why deer “act in a way that facilitates the hunter’s enterprise at the time of the encounter,” has not yet been explored in the current ethnographic context. Future studies that address this topic in nonindigenous contexts might be able to provide unprecedented contributions to the anthropological and ethnoecological literature, particularly with regard to the layers of meanings present in this type of immediate contact between humans and animal behavior, especially during hunting episodes such as those mentioned above.

### Seasonality and use of anthropogenic forests by preys and carnivores

In this section, we analyze how mammals use different features of the local landscape and its interaction with seasonality. On this subject, the *quilombola* accounts suggest an indirect relationship between anthropogenic environments (gardens and clearings) and regional carnivores, mediated by herbivores and/or omnivores that forage in these settings. According to local accounts, autumn and winter are the seasons where fruit is more abundant in the secondary forests (or fallows), “because cold is the time for fruit.”

“The little critters come into the garden to eat roots and fruit, which brings out the big cats who go where their food is…in the middle of the year, the cats all come seeking out food, so they get closer because what they eat is much closer to us“ (Renato, age 72, RCS’s field journal, 05/27/2017).

This type of account indicating the potential for traditional garden plots to attract mammals has been reported in the same *quilombola* context [22], as well as among coastal populations of São Paulo state [50]. In the broader literature, it is known that traditional agricultural systems (called itinerant gardens, slash-and-burn, or coivara cultivation) [76, 77] and the secondary forests they produce can add complexity to the landscape and impact on faunal dynamics and local hunting strategies [78, 79].

For example, as Linares [80] and Smith [81] suggested long ago, the Buglé indigenous people in Panama describe garden plots and fallow areas (secondary forests) as veritable “game gardens” because they attract many mammal species. Other examples that corroborate this include case studies on primates in Africa [82, 83], birds in the Colombian Amazon [84] and Guatemala [85] and small mammals in Mexico [86], for a broader review of this topic, see [87]. The traditional agricultural system employed by *quilombola* communities in Ribeira Valley is slash-and-burn and itinerant farming [35], which over time have produced a mosaic of secondary and mature forests in that region [34, 88].

Additionally, various local collaborators provided information potentially relevant to a better understanding of the ecology and space use patterns by this faunal group, such as the occurrence of coatis in areas with an abundance of bromeliads (especially *Vriesea* sp., Bromeliaceae), which is in agreement with their foraging in Ribeira Valley [89]; an association of the fox and raccoon with old banana groves that are still scattered throughout the landscape; and the puma’s movement pattern by relatively fixed routes but especially on the jutting mountaintops that are characteristic of the region’s topography.

Considering the feline reproductive behavior and its association with landscape use and seasonality, many locals repeated the narrative that the jaguar mates during the months of August and September.

“Around August/September, you can hear the jaguar roaring out there in the forest [the interviewee imitates the sound of a jaguar], and we see a lot of scratch marks on the ground and on the trees because they're starting their reproductive period...and the jaguars are all giving birth at the end of the year, they stay up there in the forest“ (Danilo, age 65, RCS’s field journal, 05/27/2017).

In general, jaguars can reproduce throughout the year [90, 91], with a greater concentration of reproductive activity occurring in certain periods. For example, the species’ reproductive peak in Belize was recorded between May and September [92]. The period of December to February was reported to be the most active for the species’ reproduction in Venezuela [93] and in the Brazilian Pantanal [94].

Hormonal analyses of specimens in captivity indicate that the jaguar’s ovarian activity begins in August and September [95]. These data corroborate the *quilombola* account cited above. In the environmental context of Ribeira Valley in particular, the spring season, which begins in September, was identified as the jaguar’s mating period in Carlos Botelho State Park [96], a conservation unit contiguous with the Quilombos do Middle Ribeira Valley Quilombolas Environmental Protection Area [*Área de Proteção Ambiental Quilombos do Médio Ribeira*, *APA-QMR*] and its *quilombola* territories. These data also align with the account mentioned above.

### Hunting primates: ethnoprimatology, phenomenology, and regret

Another topic that is relevant is the relationship between *quilombolas* and primates. For example, *quilombolas* have reported that when wounded by gunfire, howler monkeys and southern muriqui often use leaves, which they rub over or stuff into the wound. This is an ethnoecological record of what is known in the zoological literature as anointing or fur-rubbing behavior [97].

The local residents’ interpretation is that these primates use the leaves as a sort of “medicine” to cure the wound or at least relieve the pain it causes. In this case, the anointing behavior seems to be associated with the concept of zoopharmacognosy: the therapeutic use of substances or materials by animals suffering from some type of injury [98, 99].

Whether or not associated with zoopharmacognosy, anointing has been observed among orangutans (*Pongo pygmaeus wurmbii*) [100], capuchin monkeys (*Cebus capucinus*) [101], spider monkeys (*Ateles geoffroyi*) [102], and owl monkeys (*Aotus* spp.) [103]. Based on a preliminary survey, however, there is not yet any systematic and reliable description of this behavior among howler and southern muriqui.

Another aspect of this behavior is that when local residents discuss it, they usually also report that if the animal is not felled by the first shot and can grab the leaves and rub them on its wound, it will almost certainly not be killed. Some residents even say that if this occurs, “it’s better to go home,” relating instances in which the animal was never killed, even after having been hit by many gunshots. In addition to the empirical data related to the concept of zoopharmacognosy, these particular aspects of the narratives seem to hint at a magic dimension to this behavior in that the behavior is interpreted as rendering the animal immune to death, at least in that particular hunting episode.

This sort of magic behavior may be associated to the concept of “corpo fechado” (closed body). Varela [104], based on the classical work of Marcel Mauss [105], “A General Theory of Magic,” defines “corpo fechado” as a belief in a type of invulnerability to death created by a sort of spell or enchantment, common in human and animals. The mediation of spirits and entities of this form of enchantment is commonly combined with use of materials such as leaves, roots, and rocks. This kind of belief might take elaborated rites among humans, but it is perceived that individual animals might be able to do it with the aid of forest supernatural entities, which also may take the form of animal itself in local ontologies [9–14, 106].

Another topic related to primates is a set of reports by hunters (or former hunters) that relate traumatic experiences involving the killing of primates. These accounts tell of “near-human” behavior manifested by primates at the time they are killed. There are accounts, for example, of females who, when shot, protect their young from the fall; of offspring who clutch their wounded mothers until they can no longer bear her weight; and of mothers who display their babies to the hunter when he is preparing to take the kill shot.

Returning to the subject of animal behavior, Zeca (age 58) tells of the time "a monkey" [a female southern muriqui] he had shot pulled a baby (theretofore not seen by the hunter) from behind her back and, before falling, threw the baby up into the branches where it could grab hold and thus be saved from the fall. Zeca related this episode with great regret, saying it is one of the reasons he does not much enjoy hunting, especially monkeys (HMP’s field journal, 12/07/2018).

In the field, these stories are told with a certain dramatic tone and sense of regret, in general culminating in expressions such as “monkeys are almost people” and “it seems like a monkey used to be a person,” and “after what I saw, I never killed a monkey again.”

In this regard, it is worth noting that attributing human qualities to other animals can be associated with animist thought, which is predominant among indigenous peoples in the Amazon, the circumpolar regions of Canada and Siberia, and parts of Indonesia, among other regions [10]. This is not the case of the ethnographic context considered here. This is more an anthropocentric conception of Western origin [75], directed at a specific element of nature, in this case, primates—also observed in other nonindigenous rural populations in Brazil [106, RSM and HMP, pers. obsv. among Amazonia’s riverine populations].

The explanation for this example of anthropocentrism, particularly directed at primates, seems to lie in their physical and behavioral similarity to humans. These similarities are perceived in the course of the intimate involvement of people with these animals, mobilized especially in the daily experience of hunting. Therefore, based on this phenomenological dimension, it seems that this anthropocentric conception of these animals arises from the bodily/sensorial experience between local hunters and primates.

These ontological data can, in turn, modulate the constitution of symbolic representations about these animals [107], which still needs to be understood in this kind of ethnographic context. This is a phenomenon that has not yet received much attention in research on nonindigenous rural populations in Brazil [106]. Consideration of this kind of phenomenon may have significant implications for the development and effective implementation of management plans and environmental education programs related to this faunistic group [108, 109].

## Conclusions

In this article, we have compiled a set of local knowledge about behavior, activity period, and spatial-temporal dynamic of species hunted among *quilombolas* from Southeast Brazil. The data presented here also provide a detail ethnographic report about the nocturnal hunting method of “trepeiro,” the chases of deer and peccary with dogs, and regarding the unique behavior of monkeys during hunting episodes. Together, these accounts make up a set of qualitative and processual dimension of hunting activity of conservation and anthropological interests, especially for the context of rural communities in the Neotropics.

Local narratives about hunting and the natural history of species formed the core of the results obtained in this study, and these were related to the corresponding scientific knowledge. This approach was not based on the premise that the epistemological value of local knowledge depends on a process of validation by normative science [110]. We sought, however, to bring to light the local narratives [111] as an expression of *quilombolas*’ environmental knowledge as a value in itself. As we conducted bibliographic research on topics mentioned in local accounts, we found that local narratives were almost completely corroborated by scientific knowledge about the species considered here. We were then able to articulate these two repertoires in the body of the text, exploring precisely the consistency between them.

This exercise resulted in the construction of a comprehensive and unified narrative about aspects of the natural history of these species. Sometimes scientific knowledge corroborated local narratives, although with regard to other related species in other environmental contexts (i.e., Amazonia, United Kingdom, etc.). In other cases, the authors’ ethnographic experience with local residents added details about the ecology and behavior of species rarely mentioned in academic texts guided by a naturalistic-quantitative approach.

Although they differ in many epistemological and ontological aspects, natural scientists and members of rural communities share an empirical experience of the environment in which they find themselves, whether in the course of their research or in daily practices. A common dimension of the environmental experience [23] makes it possible for ethnobiologists to carry on toward one of the main challenges of their discipline: to promote the “meeting of minds” to which Eugene Hunn refers [16]. This shared phenomenological/cognitive basis may be at the origin of the classical correspondences between the Linnaean and local taxonomies [112–114]. The consistency between the scientific and *quilombola* narratives about the natural history of the animals discussed here seems to be a part of this same phenomenon. Furthermore, identifying points of contact and promoting communication between scientific knowledge and local repertoire is a *sine qua non* for implementing effectively any proposal for the collaborative management of natural resources [115].

Beyond the dimension of knowledge, by implementing an ethnobiological study in the field using an ethnographic approach and a phenomenological orientation [24, 40], we were able to describe the activity of hunting (in the case of HMP's field research) based on the daily experience [56] of those engaged in it. It contained elements of strategy, tactics, an acquired taste for the activity itself, and desire for social status [57]. Feelings related to euphoria (such as at the moment of the kill) and negative feelings of anxiety and regret (such as the cases involving primates) were aroused in the act of hunting and were also present in the collected accounts. Taken together, these elements point toward a multitude of human behaviors and feelings that manifest themselves mainly in the context of the action in which hunting takes place [23, 55].

It would not be very plausible to believe that such behaviors do not play an important role in modulating the practice of hunting, including where and how it takes place and the level of exploitation involved [116]. As we know, these are key variables in any quantitative model that focuses on the medium- and long-term impacts of this activity [3, 117]. Thus, we also argue that the ethnographic approach should be increasingly incorporated into studies of hunting from a conservation perspective.

Especially in Brazil, the invisibility of the role of hunting in the food sovereignty of rural communities [49], together with a fragmented and ambiguous system of norms, has made it difficult to regulate hunting in the country [118, 119]. The need for studies that convey the social practices and its concrete dimensions in which hunting takes place and the set of knowledge it embodies appears even more pressing under these circumstances. We hope that the study presented here and the methodological approach we seek to develop may contribute to a more multifaceted understanding of the practice of hunting, from the microaspects of its everyday dimensions to its many points of contact with the broader scientific literature.

## Data Availability

Not applicable

## Footnotes

1 Secondary forest that covers an abandoned garden plot as part of the itinerant agriculture system traditionally used by the region's *quilombolas*.

2 Mauro also reported the technique of drowning the deer when the river is deeper. He reported events in which he waited shoulder-deep in water to grab and drown a deer as it swam past (HMP’s field journal, 07/17/2019).
